# Recurrent Exposure to Subclinical Lipopolysaccharide Increases Mortality and Induces Cardiac Fibrosis in Mice

**DOI:** 10.1371/journal.pone.0061057

**Published:** 2013-04-09

**Authors:** Wilbur Y. W. Lew, Evelyn Bayna, Erminia Dalle Molle, Nancy D. Dalton, N. Chin Lai, Valmik Bhargava, Vincent Mendiola, Paul Clopton, Tong Tang

**Affiliations:** Cardiology Section, Department of Medicine and Research Service, Veterans Administration San Diego Healthcare System, and University of California San Diego, San Diego, California, United States of America; Albert Einstein College of Medicine, United States of America

## Abstract

**Background:**

Circulating subclinical lipopolysaccharide (LPS) occurs in health and disease. Ingesting high fatty meals increases LPS that cause metabolic endotoxemia. Subclinical LPS in periodontal disease may impair endothelial function. The heart may be targeted as cardiac cells express TLR4, the LPS receptor. It was hypothesized that recurrent exposure to subclinical LPS increases mortality and causes cardiac fibrosis.

**Methods:**

C57Bl/6 mice were injected with intraperitoneal saline (control), low dose LPS (0.1 or 1 mg/kg), or moderate dose LPS (10 or 20 mg/kg), once a week for 3 months. Left ventricular (LV) function (echocardiography), hemodynamics (tail cuff pressure) and electrocardiograms (telemetry) were measured. Cardiac fibrosis was assessed by picrosirius red staining and LV expression of fibrosis related genes (QRT-PCR). Adult cardiac fibroblasts were isolated and exposed to LPS.

**Results:**

LPS injections transiently increased heart rate and blood pressure (<6 hours) and mildly decreased LV function with full recovery by 24 hours. Mice tolerated weekly LPS for 2–3 months with no change in activity, appearance, appetite, weight, blood pressure, LV function, oximetry, or blood chemistries. Mortality increased after 60–90 days with moderate, but not low dose LPS. Arrhythmias occurred a few hours before death. LV collagen fraction area increased dose-dependently from 3.0±0.5% (SEM) in the saline control group, to 5.6±0.5% with low dose LPS and 9.7±0.9% with moderate dose LPS (P<0.05 moderate vs low dose LPS, and each LPS dose vs control). LPS increased LV expression of collagen Iα1, collagen IIIα1, MMP2, MMP9, TIMP1, periostin and IL-6 (P<0.05 moderate vs low dose LPS and vs control). LPS increased α-SMA immunostaining of myofibroblasts. LPS dose-dependently increased IL-6 in isolated adult cardiac fibroblasts.

**Conclusions:**

Recurrent exposure to subclinical LPS increases mortality and induces cardiac fibrosis.

## Introduction

Lipopolysaccharide (LPS), a glycoprotein from gram negative bacteria, activates innate immunity through toll-like receptor 4 (TLR4) [1]. Healthy individuals are exposed to circulating LPS in common conditions, including after ingestion of high fatty meals [2], [3], smoking [4], with severe exertion [5], and periodontal disease [6]. Persistently elevated LPS levels are found in chronic diseases, including type II diabetes mellitus [7], chronic infections of the respiratory, gastrointestinal, and genitourinary tracts [4], decompensated heart failure [8], metabolic syndrome, cirrhosis, alcoholic fatty liver disease and hepatitis [9]. The consequences of recurrent episodes of exposure to subclinical LPS on the heart and survival are unknown.

Subclinical LPS may have metabolic and vascular effects. Exposure to LPS from gut microbiota induces a metabolic endotoxemia that may contribute to obesity, glucose intolerance, and insulin resistance [9]. Chronic LPS may contribute to vascular inflammation and atherosclerosis [10]. Severe periodontal disease is associated with increased carotid artery intima-media wall thickness [11], [12], and impaired brachial arterial endothelial function [13] that improves 6 months after intensive periodontal therapy [14].

The heart may be a target for LPS, since cardiac cells express TLR4 [15]. Activation of the innate immune system in the heart by TLR4 has diverse effects with cardioprotective myocardial effects in the short-term, whereas sustained activation may be maladaptive [16]. The myocardial effects of recurrent subclinical activation of TLR4 is not known. Studies from this laboratory demonstrated that low levels of LPS activate cardiac myocytes to depress contractility [17], and induce apoptosis by activating the cardiac renin-angiotensin system (RAS) and angiotensin type 1 receptors (AT_1_-R) [18], [19]. Since RAS activation may lead to cardiac fibrosis [20], it was hypothesized that LPS may induce cardiac fibrosis. Recurrent exposure to subclinical LPS may have cumulative effects to alter left ventricular (LV) structure and function and decrease survival.

In order to test these hypotheses, a murine model of recurrent exposure to subclinical LPS was developed. Rodents are suitable for a chronic model because they are more resistant to sudden death after acute exposure to LPS compared with other species. Mice were injected with intraperitoneal (i.p.) LPS once a week in doses that caused no distress and with only mild transient effects on LV function and hemodynamics that resolved within 6–24 hours. Mice tolerated weekly i.p. LPS for 2–3 months with no change in activity, appetite, weight, blood pressure, or blood chemistries. However, LPS induced a dose-dependent cardiac fibrosis and increase in mortality, which are major adverse consequences of this seemingly benign subclinical condition.

## Materials and Methods

### Ethics Statement

Experiments were performed in accordance with institutional guidelines and the “Guide for the Care and Use of Laboratory Animals”, Eighth Edition published in 2011. The studies were approved by the Institutional Animal Care and Use Committee and Research and Development Committee of the VA San Diego Healthcare System. All animals were monitored daily (7 days/week) for any signs of distress, lethargy, labored breathing, anorexia, or refusal to eat or drink. If mice developed any of these symptoms or anorexia with weight loss of more than 1–2 gm over 2–3 days, mice were euthanized. Mice completing the protocol were euthanized after inducing deep anesthesia with an i.p. injection of sodium pentobarbital 50 mg/ml, and after loss of paw withdrawal, opening the chest and excising the heart by surgical dissection to induce death by exsanguination.

### Experimental Protocol

All studies were performed in male C57Bl/6 mice, greater than 2 months in age (25–30 gm) from Charles River Laboratories. Mice were injected i.p. with LPS (*Escherichia coli* 055, LPS no. B5, List Biological Laboratories, Campbell, CA) in doses of 0.1, 1.0, 10 or 20 mg/kg (volume diluted with saline in 0.5 ml). Mice were injected with 0.5 ml of saline as a control.

Echocardiography was performed with a Sonos 5500 (Philips Medical Systems, Andover, MA) with a L15–6 MHz linear transducer. Acute changes in LV function were assessed at baseline (time = 0), 6, or 24 hours after i.p. injection of LPS or saline. Chronic effects were evaluated after two and three months of weekly i.p. injections of LPS or saline. Mice were anesthetized with isoflurane 5% in 100% oxygen for one minute, and then maintained with 1% isoflurane in 100% oxygen by face mask at 1 L/minute flow rate. Standard M-mode images were recorded at the level of the papillary muscles with parasternal short-axis views. Three consecutive cardiac cycles were averaged to measure LV internal diameter at end-diastole and end-systole, fractional shortening (% change in LV diameter from end-diastole to end-systole), LV wall thickness of the interventricular septum and posterior wall at end-diastole and end-systole, percent wall thickening (from end-diastole to end-systole), and indices of LV contractility with aortic ejection time and velocity of circumferential shortening.

Mice were instrumented for telemetry to continuously record the ECG and measure heart rates. Mice were anesthetized with ketamine (100 mg/kg) and xylazine (5 mg/kg) to implant a radio transmitter (EA-F20, Data Sciences International, Inc., St. Paul, MN) into the peritoneal cavity with wires tunneled subcutaneously and sutured over the right thorax and left lower abdomen to simulate an ECG lead II [21]. Mice were allowed to recover for at least one week before receiving injections. Transmissions from the telemetry units continuously recorded the ECG, activity and body temperature on a PC using Dataquest A.R.T. software (Data Sciences International). ECG signals were sampled at 2000 Hz. Mean heart rate (moving average over 10 seconds) was recorded for 1 hour before and 8 to 24 hours after injections.

Blood pressure was measured in conscious mice trained to lie quietly in a restraining cage. A tail-cuff was placed to occlude blood flow and a volume pressure sensor probe placed distally to measure systolic, diastolic and mean blood pressure and heart rate using the Coda™ Non-Invasive Blood Pressure System (Kent Scientific, Torrington, CT).

The effects of angiotensin type 1 receptor (AT_1_-R) blockade was examined in mice with losartan added to the drinking water, beginning three days prior to injections and continued throughout the protocol. Weights and average daily water consumption were recorded for each mouse to adjust drug concentrations in the water for a dose of losartan at 20 mg/kg/day.

Cutaneous pulse oximetry was measured with a MouseOx Pulse Oximeter from Starr Life Sciences (Oakmont, PA), with MouseOx software to record oxygen saturation. Blood was collected at the time of sacrifice for a complete blood count and blood chemistry, performed by the Animal Care Program UCSD Diagnostic Laboratory.

Picosirius red staining was performed on short axis midwall LV rings that had been formalin-fixed and paraffin embedded. De-waxed sections (6 µm) were rehydrated, stained with picrosirius red (1 h). LV sections were then dehydrated using graded concentrations of ethanol and mounted in Permont. Fractional area of fibrosis was quantified using NIH image software ImageJ. LV sections were immunostained with antibodies to α-SMA and Ki67 to identify myofibroblasts and proliferating fibroblasts as previously described [22].

Quantitative reverse transcriptase-polymerase chain reaction (QRT-PCR) was used to measure expression of fibrosis, inflammatory cytokines, and hypertrophy related genes. Total RNA was extracted from LV samples, digested with RNase-free DNase, and reverse transcribed. QRT-PCR was performed and RNA equivalents normalized to simultaneously determine glyceraldehyde-3-phosphate dehydrogenase (GAPDH) mRNA levels in each sample.

Cardiac fibroblasts were isolated from adult mouse hearts using a Langendorf apparatus and digested with collagenase solution as previously described [23]. Fibroblasts from 2 hearts were combined, plated in media, and grown to 90–100% confluence and studied within two passages. Fibroblasts were starved and treated with 0.5% irradiated, low endotoxin FBS and exposed to 0, 0.1, 1.0 and 10 ng/ml LPS for 48 hours.

Statistical analyses for comparing multiple groups were performed with SigmaPlot 11 for one way and two-way repeated measures analysis of variance (ANOVA). Multiple comparisons between two or more groups were made using the Holm-Sidak Test. A mixed model analysis was performed with SPSS v.13 to evaluate blood pressure and heart rates at baseline, and their response 24 hours after injections, and temporal trends over three time periods during the protocol. A log-rank (Mantel-Cox) test was performed to evaluate Kaplan-Meier curves to detect differences in survival using GraphPad Prism v. 5. A Gehan-Breslow Wilcox test was used to detect differences between individual survival curves, using a Bonferroni method to correct for multiple comparisons. In all cases, differences were considered significant at P<0.05, with additional tests used for post-hoc analyses as noted.

## Results

### Murine model of recurrent subclinical LPS exposure

Mice were injected with i.p. saline or LPS (0.1, 1.0, 10 or 20 mg/kg) with no signs of distress or anxiety. Mice tolerated weekly injections of all doses of LPS without distress, change in activity, appearance, appetite or body weight. Injecting mice with 10 mg/kg LPS i.p. produced serum LPS levels of 57.7±4.8 ng/ml (± SEM, n = 6) at 24 hours, which decreased to 0.1±0.2 ng/ml by 7 days (n = 6, P<0.0001).

### Recurrent subclinical LPS increases mortality

**Figure 1:**
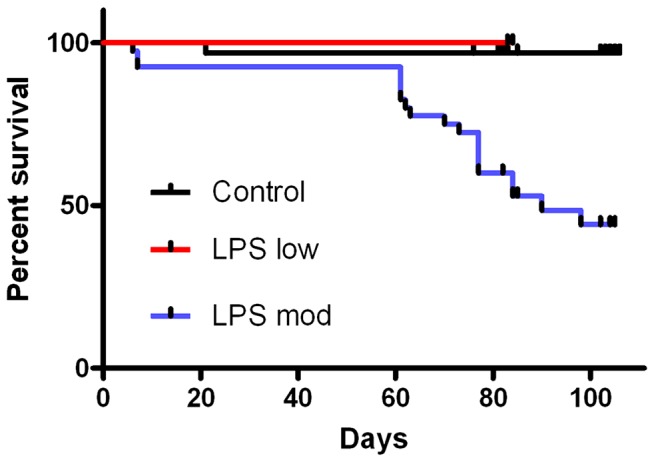
Survival with weekly injections of saline or LPS.

Weekly exposure to subclinical LPS caused a dose-dependent increase in mortality. There was no differences between 0.1 and 1 mg/kg LPS, nor between 10 and 20 mg/kg LPS, so data were combined as low and moderate LPS doses, respectively. Figure 1 shows that weekly i.p. injections of LPS had dose-dependent effects on survival (P<0.0001). Low dose LPS (0.1–1.0 mg/kg/week) did not affect survival, which was similar to the saline control group, but moderate dose LPS (10–20 mg/kg/week) decreased survival from 60 to 90 days with a reduction in median survival to 90 days. Survival was lower with moderate dose LPS compared with low dose LPS (P<0.005) or saline (P<0.0001). Deaths occurred throughout the 7 day period and were unrelated to the timing after injection (mean time 3.8±0.4 days).

There was no significant difference in body weight between groups at any time during the protocol. At 8 weeks, body weights were 29.0±0.5 g (n = 31) in the control group, 27.3±0.6 g (n = 16) with low dose LPS, and 28.3±0.3 g (n = 35) with moderate dose LPS (P = ns). At 12 weeks, body weights were 29.7±0.5 g (n = 31), 28.9±0.5 g (n = 16) and 28.7±0.2 g (n = 24), respectively (P = ns). There was no difference in body weights at 8 weeks of mice treated with moderate dose LPS that would eventually die (28.7±0.5 g, n = 14), compared with mice that survived to the end of the protocol (28.2±0.3 g, n = 14).

### Left ventricular size and systolic function preserved after recurrent LPS

**Figure 2:**
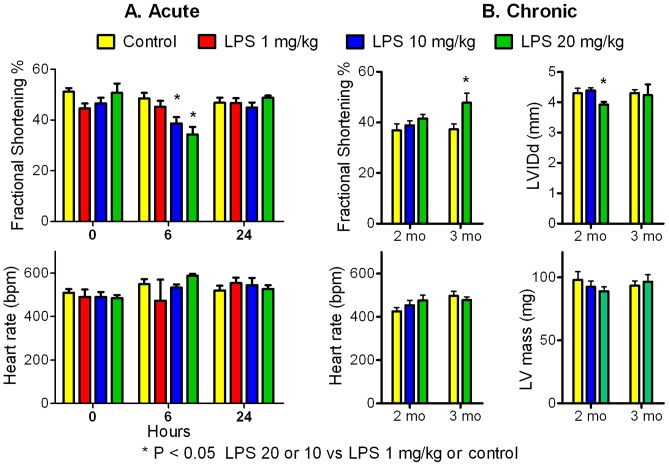
Acute and chronic effects of LPS on left ventricular (LV) size and function by echocardiography. Panel A: LV fractional shortening and heart rate (beats per minute, bpm) measured before (0 hours), and 6 and 24 hours after i.p. injection of saline (control), LPS 1, 10, or 20 mg/kg (each bar, mean+SEM, n = 6). There was a mild decrease in LV fractional shortening after 6 hours with LPS 10 and 20 mg/kg (*P<0.05 vs LPS 1 mg/kg or control) that resolved by 24 hours. Panel B: LV fractional shortening, heart rate, LV internal diameter in diastole (LVIDd), and LV mass measured after 2 months of weekly i.p. injections of saline (n = 11), LPS 10 mg/kg (n = 8) and LPS 20 mg/kg (n = 12), and after 3 months of weekly i.p. injections of saline (n = 13) or LPS 20 mg/kg (n = 5). There were a few minor changes with LPS 20 mg/kg (*P<0.05 compared with control), but no significant decrease in LV function, LV dilation, or change in LV mass.

Subclinical LPS had minimal effects on LV size and systolic function when measured acutely (0, 6, 24 hours) or chronically (2 and 3 months) by echocardiography. Figure 2, Panel A shows a mild decrease in LV fractional shortening 6 hours after moderate doses of LPS (10 and 20 mg/kg, P<0.05), with full recovery by 24 hours. There was no change in LV fractional shortening with low dose LPS (1 mg/kg). Heart rates did not differ at any time with any dose of LPS. There were no changes in LV diameter, wall thickness, % wall thickening of the interventricular septum and posterior wall, or indices of LV contractility (velocity of circumferential shortening and aortic ejection time) at any time with any LPS dose (not shown).

Figure 2, Panel B shows minor changes in echocardiographic measures after 2 and 3 months of weekly i.p. LPS 20 mg/kg/week compared with control (decrease in LVIDd at 2 months, increase in LV fractional shortening at 3 months, P<0.05), with no change in heart rate or LV mass at any time. Thus, there was no evidence of decreased LV systolic function or LV dilation after 2 to 3 months of weekly injections of LPS.

### Acute and chronic hemodynamics effects of subclinical LPS

Five mice were instrumented for telemetry and monitored continuously after injection of LPS 10 mg/kg i.p. Heart rate (HR) increased acutely from a baseline of 477±8 (SEM) beats per minute (bpm) to a peak of 724±8 bpm, and then returned to baseline after 6.0±0.6 hours. Mice were injected once a week for 6 weeks with LPS 10 mg/kg i.p. with similar baseline and peak HR responses each week.

**Figure 3:**
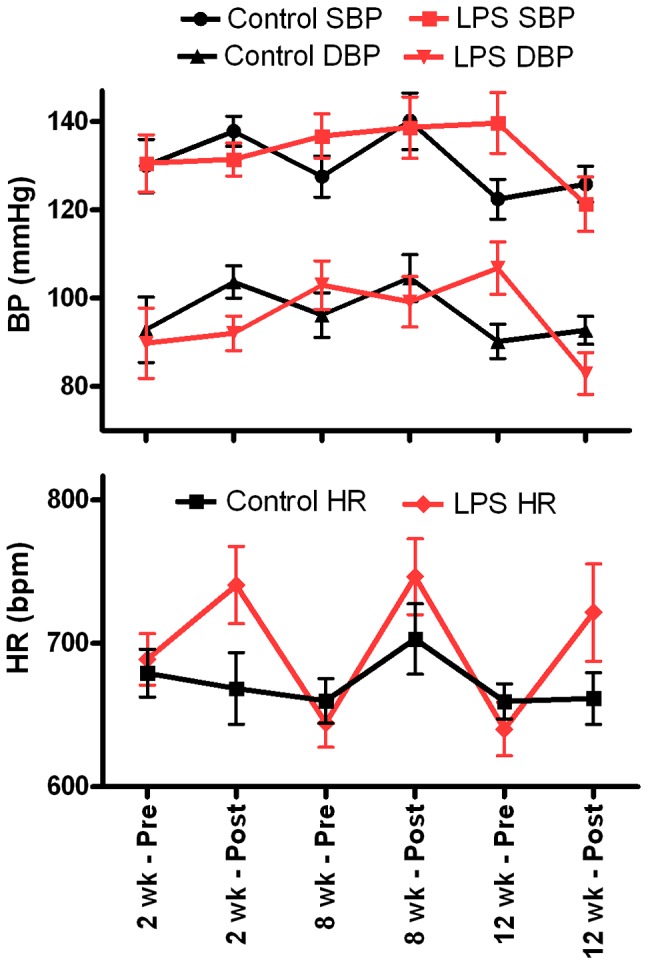
Hemodynamics with weekly injections of LPS for 12 weeks. Tail cuff systolic and diastolic blood pressure (SBP, DBP) and heart rate (HR, beats per minute, bpm) measured in conscious mice before (Pre) and 24 hours after (Post) weekly i.p. injections of saline (n = 7) or LPS (20 mg/kg, n = 6). Data (mean ± SEM) averaged over three weeks at 2 wk (1–3 weeks), 8 wk (7–9 weeks) and 12 wk (weeks 11–13). Hemodynamics were stable with no significant trends from 2 to 12 weeks. There was an interaction between LPS and the Pre-Post injection for SBP (P = 0.044), DBP (P = 0.015) and HR (P = 0.011) (mixed model analysis) reflecting small but consistent differences in HR and BP responses 24 hours after LPS compared with saline.

Recurrent exposure to LPS did not alter baseline hemodynamics nor hemodynamic responses to LPS. Figure 3 shows tail cuff blood pressure (BP) and HR measurements in conscious mice before and 24 hours after weekly i.p. injections of saline or LPS at 2, 8 and 12 weeks. There was no significant difference in baseline systolic BP, diastolic BP, or HR between LPS and saline, and no change over time. There was a significant interaction between LPS and change in BP and HR after 24 hours, that reflect small differences in hemodynamic responses to LPS compared with saline (i.e. higher HR and lower BP at 24 hours); these changes were small with no change in hemodynamic responses to LPS from 2 to 12 weeks.

### Recurrent subclinical LPS had minimal effects on blood counts and chemistries

Four groups of mice were given i.p. saline (n = 7), 0.1 mg/kg LPS (n = 8), 1 mg/kg LPS (n = 8), or 10 mg/kg LPS (n = 7), once a week for 11–12 weeks. Oximetry performed under isoflurane anesthesia after 11–12 weeks did not differ between groups. Arterial O_2_ saturation was 93.3±2.3% in the saline group, 93.7±1.9% with LPS 0.1 mg/kg/week, 89.9±1.4% with LPS 1 mg/kg/week, and 87.4±1.5% with LPS 10 mg/kg/week (P = ns).

Table 1 show blood counts and chemistries measured after 11–12 weeks (7–9 days after last injection). LPS did not affect total white blood cells (WBC), segmented neutrophils, monocytes or eosinophils. LPS dose-dependently increased platelets, and decreased red blood cell (RBC) counts and hemoglobin, but these mostly remained within normal ranges. LPS had no significant effects on electrolytes, renal or liver function. Alkaline phosphatase increased slightly, but remained within normal limits.

**Table 1:** Complete blood counts and chemistries.

Measure (normal values)	Saline	LPS 0.1	LPS 1.0	LPS 10	P value
**WBC×1000 (5–16/mm^3^)**	2.14±0.18	2.82±0.19	3.18±0.43	2.48±0.24	ns
**Bands**	26±6	29±12 †	69±18	121±21 *	<0.001
**Segs (600–6100)**	351±39	420±98	667±156	630±119	ns
**Lymphs (3300–6640)**	1656±143	2253±202	2240±311	1552±155	0.046
**Monocytes (0–1500)**	56±14	68±26	132±34	131±36	ns
**Eosinophils (0–510)**	46±13	49±16	76±45	50±14	ns
**Platelets×1000 (500–1500)**	802±39	995±42 * †	1104±53 *	1220±58 *	<0.001
**RBC×10^6^ (6.7–13/mm^3^)**	8.3±0.3	8.0±0.2 †	7.4±0.2 * †	6.2±0.2 *	<0.001
**Hemoglobin (10–20 g/dl)**	13.0±0.3	12.7±0.3 †	11.8±0.3 * †	9.8±0.2 *	<0.001
**Glucose (90–192 mg/dl)**	301±20	276±8	281±21	238±12	ns
**BUN (18–29 mg/dl)**	22.1±1.2	25.3±1.1	22.6±1.0	23.9±1.0	ns
**Creatinine (0.2–0.8 mg/dl)**	0.51±0.14	0.46±0.11	0.27±0.05	0.41±0.06	ns
**Total protein (3.6–6.6 g/dl)**	5.16±0.11	5.53±0.08	5.43±0.05	5.44±0.05	0.013
**Albumin (2.5–4.8 g/dl)**	3.01±0.11	3.28±0.05	3.17±0.06	3.10±0.07	ns
**Globulin (g/dl)**	2.11±0.13	2.25±0.07	2.29±0.06	2.34±0.05	ns
**Sodium (126–182 mEq/L)**	144±1	148±1	147±1	147±1	ns
**Potassium (4.7–6.4 mEq/L)**	5.66±0.15	5.80±0.17	5.87±0.35	5.63±0.24	ns
**Calcium (5.9–9.4 mg/dl)**	9.66±0.09	9.83±0.11	9.77±0.10	9.83±0.13	ns
**Bilirubin Total (0.1–0.9 mg/dl)**	0.19±0.01	0.18±0.02	0.19±0.01	0.17±0.02	ns
**SGPT (ALT) (28–132 U/l)**	25.0±1.8	35.0±3.9	31.0±2.4	32.3±2.8	ns
**Alkaline Phos (62–209 U/l)**	53.9±2.2	64.0±2.7 †	69.1±5.5 †	93.1±7.7 *	<0.001
**Amylase (1691–3615 U/l)**	900±18	905±29	901±67	738±69	ns
**Phosphorus (6.1–10 mg/dl)**	8.76±0.43	8.44±0.31	8.26±0.28	8.46±0.15	ns

Data mean ± SEM for saline control group (n = 7), LPS 0.1, 1.0, and 10 mg/kg/week (n = 8 for each LPS dose). P values for one way ANOVA, ns = not significant.

* P<0.05 vs saline;

† P<0.05 vs LPS 10 mg/kg/week with multiple comparisons by Holm-Sidak method.

### Recurrent LPS and cardiac arrhythmias

To evaluate if cardiac arrhythmias develop with recurrent LPS, mice were instrumented for telemetry and monitored after weekly injections of saline or LPS (10 or 20 mg/kg/week). No significant arrhythmias were observed in two saline and two LPS treated mice that survived for 90 or more days. Telemetry tracings were available at the time of death in three LPS-treated mice. There were no significant arrhythmias in the days prior to death, but arrhythmias were observed within the last few hours before death. Two examples are shown.

**Figure 4:**
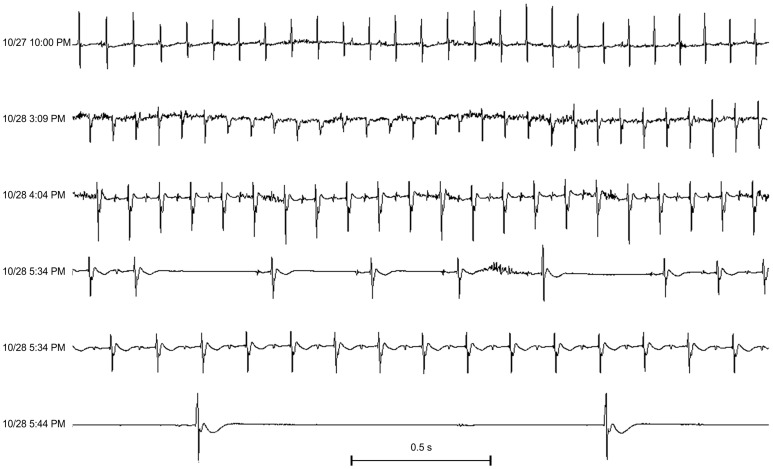
Bradycardia and high grade block develop just prior to death after weekly LPS. Telemetry tracings from a mouse that died 57 days after weekly LPS 20 mg/kg i.p. Each strip is 2.5 seconds. The top panel shows the baseline ECG at 10 pm, 18 hours prior to death, with a heart rate of 640 beats per minute (bpm). The mouse had normal activity. The remaining panels show telemetry tracings during last two hours before death. At 3:09 pm, there was a junctional tachycardia at 720 bpm with a shift in the QRS axis, consistent with increased automaticity from another region or fascicle. One hour later at 4:04 pm, sinus rhythm returned (with p waves) at 540 bpm. However 1.5 hours later at 5:34 pm, episodes of high grade block occurred with sustained bradycardia at 384 bpm, then 10 minutes later at 5:44 pm progressive high grade AV block and an idioventricular escape rhythm just prior to death.

Figure 4 shows telemetry tracings from a mouse that died 57 days after weekly LPS 20 mg/kg i.p. This mouse had normal LV systolic function with a 42% fractional shortening measured by echocardiography on day #48. There was no change in activity noted on the day prior to death (day #56). There was a normal rhythm at 640 bpm on ECG 18 hours prior to death (top panel). Two hours prior to death, a junctional tachycardia at 720 bpm developed, with a return to sinus rhythm at 540 bpm, and then in the final minutes before death, there was a progressive bradycardia with episodes of high grade atrioventricular (AV) block, and an idioventricular escape rhythm.

**Figure 5:**
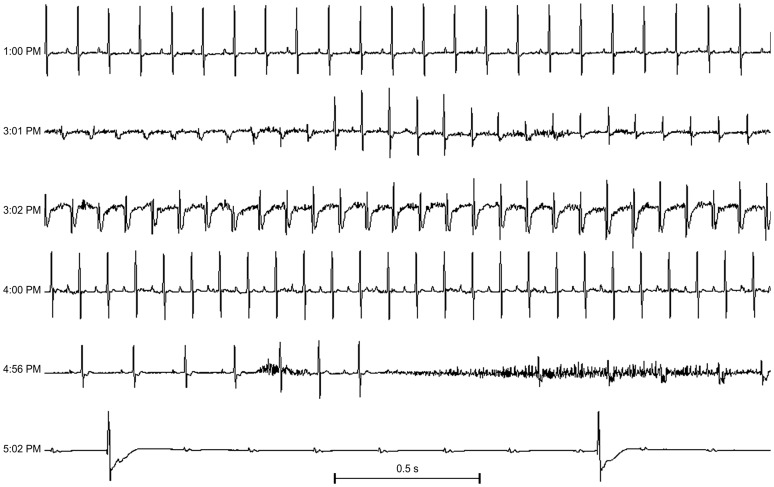
Recurrent LPS and arrhythmias prior to death. Telemetry tracings from a mouse that died 39 days after weekly LPS 10 mg/kg i.p. Each strip is 2.5 seconds. The mouse was in sinus rhythm 552 beats per minute (bpm) at baseline at 1:00 p.m. Two hours later, an accelerated idioventricular rhythm (AIVR) or slow ventricular tachycardia (VT) with fusion complexes with sinus beats developed (3:01 pm), with an AIVR or slow VT at 648 bpm at 3:02 pm. The mouse returned to sinus rhythm at 625 bpm at 4:00 pm. One hour later, there was sinus exit block with slow idioventricular escape rhythm (4:56 pm), and then high degree AV block (5:02 pm) shortly before death.

Figure 5 shows telemetry data from a mouse that died 39 days after LPS (10 mg/kg/week i.p). There was sinus rhythm at baseline. Two hours prior to death, an accelerated idioventricular rhythm (AIVR) or slow ventricular tachycardia (VT) developed. There was an initial return to sinus rhythm, but then sinus exit block developed with a slow idioventricular escape rhythm, followed by high degree AV block and then death.

### Recurrent exposure to subclinical LPS induces cardiac fibrosis

Hearts were harvested from mice treated with saline (n = 24), low dose LPS (0.1–1.0 mg/kg, n = 16) and moderate dose LPS (10–20 mg/kg, n = 23) weekly for 12–15 weeks. Table 2 shows there was no significant difference between groups for body weight, whole heart weight, left ventricle (LV) weight, or cardiac weights normalized to body weight.

**Table 2:** Heart and body weights.

	Saline	LPS low dose	LPS moderate dose
**Body weight (BW) (g)**	30.25±0.73	28.91±0.46	28.43±0.33
**Whole heart weight (HW) (g)**	0.131±0.003	0.126±0.002	0.128±0.004
**Left ventricle (LV) weight (g)**	0.103±0.002	0.102±0.002	0.096±0.002
**HW/BW×1000**	4.35±0.07	4.36±0.07	4.52±0.02
**LV weight/BW×1000**	3.42±0.07	3.55±0.06	3.33±0.07

Data mean ± SEM for saline control (n = 24), LPS low dose (0.1–1.0 mg/kg/week, n = 16), or LPS moderate dose (10–20 mg/kg/week, n = 23). BW = body weight, HW = heart weight, LV = left ventricle. None of the weights differed significantly between groups (P = ns, one way ANOVA).

**Figure 6:**
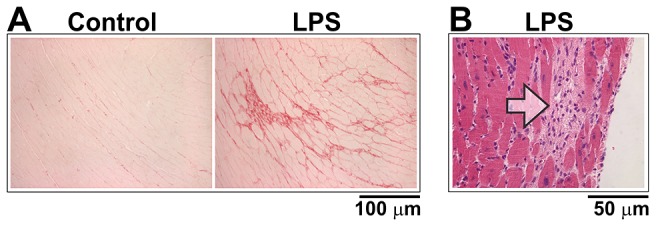
Cardiac fibrosis induced by weekly subclinical LPS. Panel A. Picrosirius staining was greater in a mouse injected with LPS 10 mg/kg/week for 12 weeks, compared with a control mouse injected weekly with saline. Panel B. H & E staining showed evidence of myofiber loss with replacement by fibrosis (arrow) after 15 weeks of LPS 20 mg/kg/week.

Hearts were sectioned and stained. Figure 6 Panel A shows an example of increased picrosirius staining in a mouse injected with LPS 10 mg/kg/week for 12 weeks, compared with a mouse injected with saline once a week for 12 weeks. Panel B shows an example of fibrosis with H & E staining after 15 weeks of LPS 20 mg/kg/week. There was no evidence of infiltration by mast cells when stained with toluidine blue (not shown).

**Figure 7:**
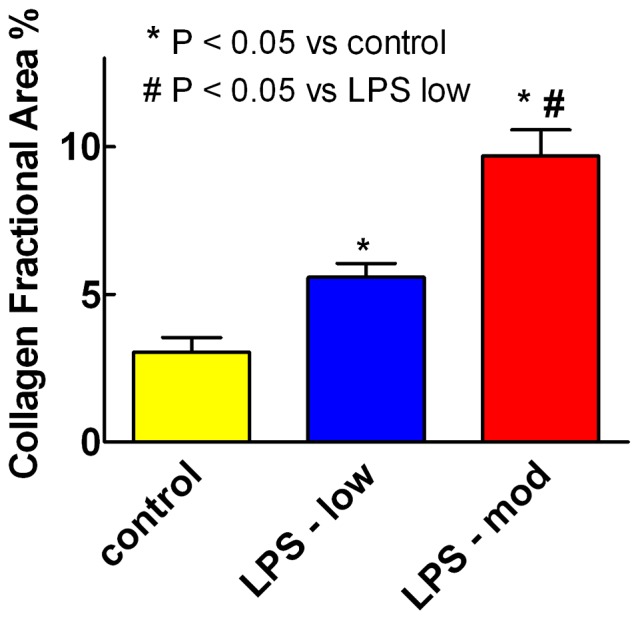
LPS dose-dependently increased percent collagen fraction area of the left ventricle. Percent collagen fraction area (mean+SEM) measured by picrosirius staining of the left ventricle increased 12–15 weeks after weekly i.p. injections of low dose LPS (0.5±0.1 mg/kg, n = 15) or moderate dose LPS (12±1 mg/kg, n = 15) compared with saline (control, n = 17) (P<0.001, one way ANOVA). There was a greater increase with moderate dose LPS than with low dose LPS (P<0.05).

Figure 7 shows LPS induced a dose dependent increase in percent collagen fraction area of the left ventricle after 12–15 weekly i.p. injections of LPS compared with saline (P<0.001, ANOVA). There was a greater increase with moderate dose LPS (12±1 mg/kg/week) compared with low dose LPS (0.5±0.1 mg/kg/week), with each LPS dose greater than with saline (P<0.05).

**Figure 8:**
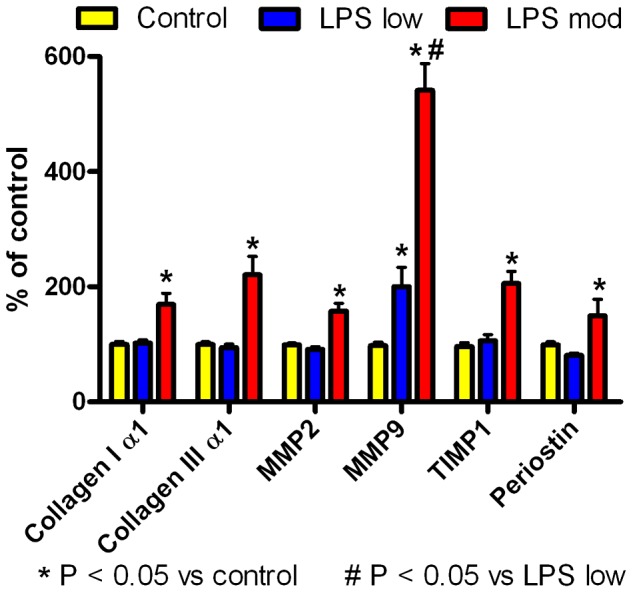
LPS dose-dependently increased expression of fibrosis-related genes in the left ventricle. Expression of fibrosis-related genes in the left ventricle measured by QRT-PCR (mean+SEM) after 12–15 weeks of weekly i.p. injections of moderate dose LPS (12±1 mg/kg, n = 15), low dose LPS (0.5±0.1 mg/kg, n = 12) or saline (control, n = 17). Collagen 1α1, collagen IIIα1, MMP2, MMP9, TIMP1, and periostin increased with moderate dose LPS compared with low dose LPS or saline (P<0.05). Low dose LPS increased MMP9 compared with saline (P<0.05).

This was associated with a LPS dose-dependent increase in LV expression of fibrosis-related genes in Figure 8. There was an increase in collagen 1α1, collagen IIIα1, MMP2, MMP9, TIMP1, and periostin with moderate dose LPS (12±1 mg/kg/week) compared with low dose LPS (0. 5±0.1 mg/kg/week) or saline controls (P<0.05). Low dose LPS increased MMP9 compared with control (P<0.05), but not the other fibrosis related genes. There was no significant difference between either dose of LPS and saline controls for expression of hypertrophy related genes, including ANF, α-SK actin, α-MHC, GATA4, or FHL1 (data not shown), which is consistent with the lack of change in LV or heart weights with LPS (Table 2).

**Figure 9:**
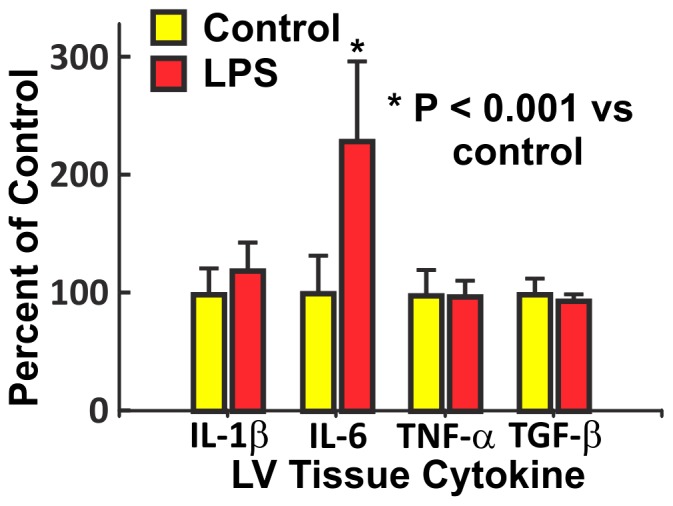
LPS increased IL-6 expression in the left ventricle. Expression of the cytokines IL-1β, IL-6, TNF-α and TGF-β (mean+SEM percent of control) were measured in the left ventricle after 3 months of LPS (20 mg/kg/week i.p, n = 5) or saline (n = 6). There was a significant increase in IL-6 (P<0.001), but no significant change in IL-1β, TNF-α, or TGF-β.

The effects of LPS on LV tissue expression of cytokines were measured by QRT-PCR. Figure 9 shows LPS (20 mg/kg/week i.p, n = 5) increased IL-6 after 3 months compared with saline (n = 6) (P<0.001), but there was no difference in expression of IL-1β, TNF-α, or TGF-β.

The potential role of fibroblasts were evaluated in LV tissue sections. There was increased myofibroblasts immunostained with α-SMA after 12 weeks of i.p. LPS 10 mg/kg/week i.p. (165±8 cells/mm^2^) compared with i.p. saline once a week (119±8 cells/mm^2^, P<0.01, n = 4 each group). There was no significant difference in Ki67 immunostaining of proliferating fibroblasts with LPS (87±10 cells/mm^2^) compared with saline (84±10 cells/mm^2^, P = ns).

**Figure 10:**
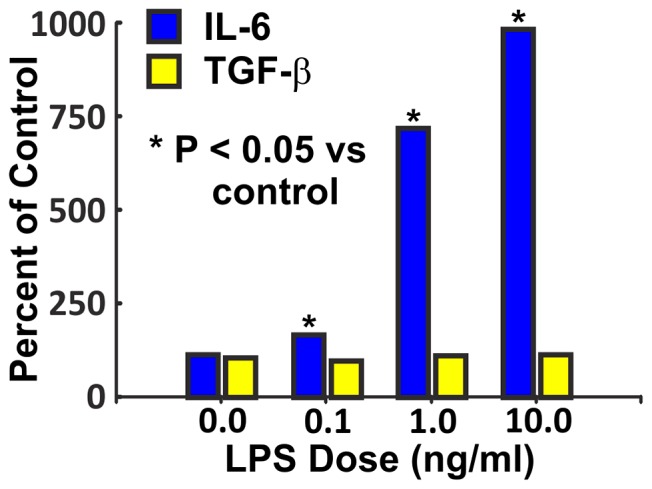
LPS increased IL-6 expression in isolated adult cardiac fibroblasts. Isolated adult cardiac fibroblasts were exposed to vehicle or LPS in concentrations of 0.1, 1.0, or 10 ng/ml for 48 hours. There was a dose dependent increase in IL-6 (P<0.001, ANOVA, each bar mean+SEM, n = 4). All LPS doses were higher than control and increased significantly with each increment in dose (P<0.05). LPS caused no significant change in TGF-β.

To evaluate if LPS directly activate cardiac fibroblasts, adult cardiac fibroblasts isolated from the left ventricle were exposed to LPS in doses of 0.1, 1.0 or 10 ng/ml for 48 hours. Figure 10 shows that LPS induced a dose-dependent increase in IL-6, but not TGF-β consistent with in vivo results after 3 months of LPS in Figure 9.

### LPS induced cardiac fibrosis not attenuated by angiotensin type 1 receptor inhibitor

It was hypothesized that LPS may induce recurrent episodes of apoptosis to induce cardiac fibrosis. This was based on prior studies showing that low levels of LPS induce cardiac apoptosis by activating cardiac RAS, which is blocked by the AT_1_ receptor inhibitor losartan [18]. Four groups of mice (n = 5–7 per group) were injected weekly with i.p. saline or LPS 10 mg/kg, with or without the AT_1_-R inhibitor, losartan (20 mg/kg/day, added to drinking water three days prior to the first injection, and continued throughout the protocol). Mice were sacrificed after 15 weeks (6–7 days after the last injection).

After 14 weeks, systolic/diastolic BP was 135±5/105±6 mmHg in the saline control group, 136±5/109±6 mmHg with LPS, 125±7/97±6 mmHg with losartan, and 142±10/115±9 mmHg in the LPS+losartan group (P = ns between groups for systolic BP, diastolic BP and mean BP). There were differences in survival between groups (P = 0.03), which at 15 weeks was 100% with saline, 90% with losartan, 80% with LPS, and 50% with LPS+losartan. Survival did not differ between mice treated with LPS compared with LPS+losartan (P = 0.27).

**Figure 11:**
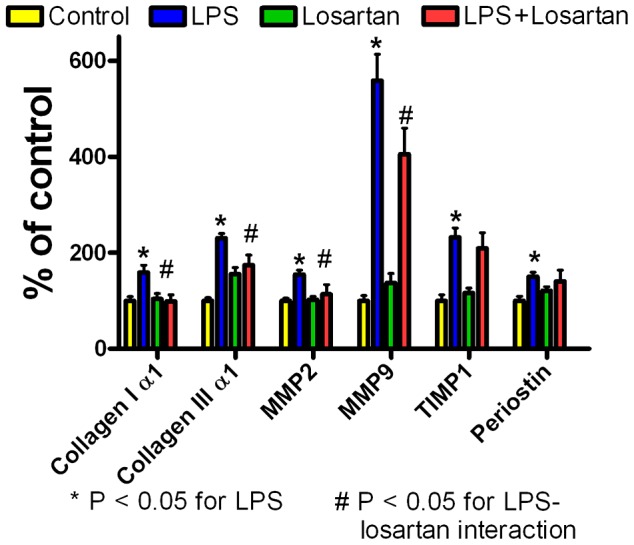
LPS activation of fibrosis-related genes attenuated by AT_1_R inhibitor losartan. Expression of fibrosis-related genes in the left ventricle measured by QRT-PCR (mean+SEM) after 15 weeks of weekly i.p. injections of saline (control, n = 7), LPS (10 mg/kg, n = 6), saline with losartan (20 mg/kg/day in drinking water) (n = 6), or LPS with losartan (n = 5). LPS increased LV expression of fibrosis-related genes including collagen Iα1, collagen IIIα1, MMP2, MMP9, TIMP1 and periostin (P<0.05, 2 way ANOVA). Adding the AT_1_R inhibitor losartan to the drinking water prevented LPS-induced increases in collagen Iα1 and MMP2 and attenuated LPS-induced increases in collagen IIIα1 and MMP9 (P<0.05 for interaction between LPS and losartan), but had no effect on TIMP-1 or periostin. Losartan alone had no effect.

Figure 11 shows losartan treatment attenuated or prevented LPS activation of several fibrosis-related genes, including collagen Iα1, collagen IIIα1, MMP2, and MMP9. Losartan alone had no effect. There was a significant interaction between LPS and losartan for these genes (P<0.05). Losartan had no effect on LPS-induced increases in TIMP-1 or periostin.

**Figure 12:**
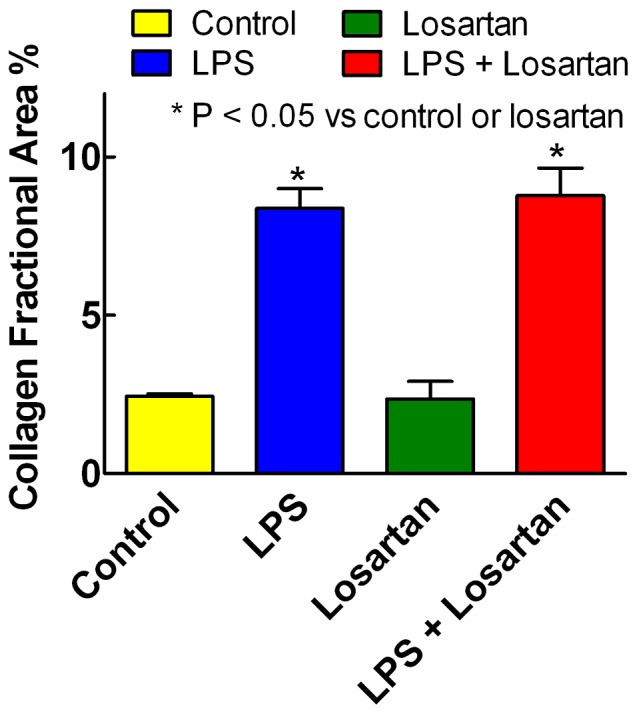
AT_1_R inhibitor losartan does not attenuate LPS-induced cardiac fibrosis. Percent collagen fraction area in the left ventricle by picrosirius red staining (mean+SEM, n = 5) increased with LPS (P<0.001, 2 way ANOVA). LPS effects were not affected when the AT_1_R inhibitor losartan was added to the drinking water (P = 0.80). Losartan alone had no effect. There was no significant interaction between LPS and losartan (P = 0.70).

Although losartan attenuated LPS activation of several fibrosis-related genes, losartan had no effect on LPS-induced cardiac fibrosis. Figure 12 shows a significant increase in LV collagen fraction area with LPS (P<0.001), which was unaffected by the addition of losartan. In contrast to several fibrosis-related genes, there was no significant interaction between LPS and losartan on cardiac fibrosis.

Other RAS mediated pathways associated with fibrosis were not affected by LPS nor losartan, with no change in LV expression of renin, angiotensin converting enzyme 2 (converts angiotensin II to angiotensin 1–7), or mas (receptors activated by angiotensin 1–7) (not shown).

## Discussion

The major findings of this study are that recurrent exposure to subclinical LPS increases mortality and induces cardiac fibrosis. Weekly exposure to LPS in doses with mild, transient effects that resolved within hours, increased mortality after 2–3 months. Deaths occurred unexpectedly, as mice appeared with normal activity, appetite, weight, hemodynamics, and with normal measures of LV size, LV function, oximetry, blood counts and chemistries. Subclinical LPS induced dose-dependent cardiac fibrosis after 3 months, even with low LPS doses that did not affect survival. This has significant implications since recurrent exposure to subclinical LPS occurs commonly, yet has not been previously considered a risk factor for causing cardiac fibrosis or increasing mortality.

A model was needed to study recurrent exposure to subclinical LPS. Subclinical LPS has been injected into normal human volunteers in several studies, but it is not ethical to expose humans to repeated doses of LPS with unknown consequences. A preclinical animal model was needed, but some animals do not tolerate exposure to low doses of LPS without acute effects including death. This may occur even after initially tolerating a few doses of LPS, making these animals unsuitable for studying more chronic effects. There is a 10,000 fold range in LPS doses for inducing 50% mortality in different species. Humans, rabbits, calves and sheep are more sensitive to LPS than guinea pigs, hamsters or dogs, whereas rats and mice are least sensitive to LPS [24]. The capacity for rodents to tolerate recurrent LPS challenges without acute lethality makes them suitable for a chronic model.

There is a 500 fold difference in the 50% lethal dose for LPS in mice compared with humans. Similarly, there are species-dependent differences in dose effects of subclinical LPS. It requires a 250 fold higher LPS dose to produce a similar increase in plasma IL-6 in C57Bl/6 mice as in humans [25]. Species-dependent differences in LPS resistance are largely related to serum factors, rather than intrinsic differences in cell response [24]. In vitro, cellular responses to LPS are markedly attenuated in the presence of mouse serum compared with human serum.

Injecting 10 mg/kg LPS i.p. produced serum LPS levels of 58 ng/ml at 24 hours. Since it requires 250–500 fold higher LPS doses to produce comparable effects in mice as in humans [24], [25], a LPS level of 58 ng/ml in the mouse may be comparable to LPS levels of 0.1–0.2 ng/ml (116–232 pg/ml) in humans. This is within the range of subclinical LPS levels found in several human conditions [9].

The experimental protocol minimized confounding factors such as LPS tolerance, which develops rapidly (e.g. 24–72 hours) after LPS exposure to decrease the responsiveness to LPS for several days. There were no priming effects, where initial exposure to LPS enhanced the response to subsequent LPS exposure. Mice were allowed to recover fully in-between weekly doses of LPS, with neither cumulative nor attenuated responses to subsequent LPS exposure. Measurements were performed 6–7 days after an injection to measure the chronic effects of recurrent LPS exposure separate from the acute effects that occur hours after injecting LPS.

The subclinical phenotype of this model was evident with only minimal or subtle effects that did not persist. LPS caused transient changes in hemodynamic and LV function that resolved after 6–24 hours. Weekly injections of LPS for 2–3 months were well tolerated with no change in activity, appetite, weight, hemodynamics, LV size or function. Recurrent LPS did not alter oxygen saturation, blood chemistries, renal or hepatic function. Despite this seemingly benign tolerance to weekly subclinical LPS, cardiac fibrosis developed with an increase in mortality after 2 to 3 months.

The mechanism for subclinical LPS to increase mortality is unknown. Arrhythmias or high grade blocks may cause sudden cardiac death. Chronic arrhythmias were not observed in surviving mice, or in the days preceding death. Terminal arrhythmias and high grade blocks were observed in the minutes to hours before death. Cardiac fibrosis may contribute to arrhythmias and high grade conduction blocks. It is unknown if either cardiac fibrosis or arrhythmias were the cause for sudden death, or merely associated findings. Cardiac fibrosis may develop with low dose LPS, which does not affect survival.

Previous studies have shown that LPS exacerbates preexisting abnormalities, but have not shown that subclinical LPS alone can cause fibrosis without prior injury. In the liver, hepatic fibrosis induced by bile duct ligation or with chemical insults (CCl_4_ or thioacetamide), is promoted by LPS from gut bacteria that activates hepatic stellate cells [26]. In the kidney, renal fibrosis induced by urethral obstruction is promoted by TLR4 activation [27]. In the heart, myocarditis induced inflammatory fibrosis is exacerbated by LPS [28]. Thus, LPS accelerates or exacerbates fibrosis induced by pre-existing injuries to the liver, kidney and heart. A novel finding in the current study is that subclinical LPS can induce cardiac fibrosis as a primary effect, without a preceding insult or injury to the heart.

The mechanisms for LPS-induced cardiac fibrosis are unknown. It was postulated that LPS activates cardiac RAS to induce fibrosis, since RAS activation is a well known cause of cardiac fibrosis [20], and LPS activates TLR4, RAS and AT_1_-R in the heart and cardiac myocytes [18], [19], [29]. It is well established from clinical trials that AT_1_-R blockers effectively inhibits fibrosis, improves LV remodeling and function and prolongs survival [30], [31]. However, losartan did not prevent LPS-induced fibrosis, nor did it reduce mortality with LPS. Since a single injection of LPS increases cardiac apoptosis for 1–3 days, which is inhibited by losartan [18], it is unlikely that LPS induces recurrent episodes of apoptosis as a mechanism for cardiac fibrosis.

Losartan attenuated LPS-induced activation of several fibrosis-related genes, including collagen Iα1, collagen IIIα1, MMP2, and MMP9, but did not attenuate LPS activation of TIMP1 or periostin. It may require broader effects on several extracellular matrix proteins to shift the balance between matrix metaloproteinases (MMPs) and tissue inhibitors of MMP (TIMPs) to prevent LPS induced fibrosis [32]. Periostin is an extracellular matrix protein that is not normally secreted in adults. Periostin is expressed in response to injury and plays a role in the fibrotic response [33]. It is unclear if periostin contributes to, or is a biomarker for LPS-induced fibrosis.

LPS did not activate other RAS mediated pathways associated with fibrosis, including renin, angiotensin converting enzyme 2, or mas receptors. Collectively, these results along with the losartan studies indicate that LPS-induced fibrosis is not primarily mediated by activation of RAS, AT_1_R, or other RAS-mediated pathways.

LPS increased cardiac expression of IL-6, which plays a role in fibrosis in multiple organs [34]. IL-6 induces cardiac fibrosis by causing differentiation of fibroblasts to myofibroblasts with increased collagen production [35]. In support of this mechanism, LPS increased the density of LV myofibroblasts measured by α-SMA immunostaining. LPS directly activated isolated cardiac fibroblasts in vitro to increase expression of IL-6. Thus fibroblasts may be the cardiac source for increased expression of IL-6 with LPS.

LPS did not activate IL-1β, TNF-α, or TGF-β. These negative results, along with the lack of involvement of RAS, indicate that LPS induces cardiac fibrosis by unique mechanisms that differ from other pathological conditions associated with cardiac fibrosis.

LPS induced cardiac fibrosis and increased mortality. Deaths were unexpected without any preceding change in activity, appetite, weights, cardiac function, pulmonary, liver, or renal function. Cardiac fibrosis can cause arrhythmias which were observed in the last few hours immediately preceding death. Although arrhythmias are a common cause of sudden, unexpected death, the evidence linking cardiac fibrosis and/or arrhythmias with death remains circumstantial. Further studies are needed to identify the mechanisms of LPS-induced fibrosis to determine if these factors can be dissociated. The cardiac fibrosis induced by LPS is unique, and does not involve other common pathways, such as RAS, apoptosis, or TGFβ. Unique therapeutic targets need to be identified to abrogate LPS-induced fibrosis to determine if this prevents arrhythmias and/or death.

The results from this study have broad clinical relevance as recurrent or chronic exposure to subclinical LPS occurs commonly. Circulating LPS increases after ingestion of a high fat, high carbohydrate diet [36]. In healthy subjects, transient exposure to LPS causes inflammation associated with insulin resistance [37]. This supports the concept of metabolic endotoxemia, where a high fat diet increases circulating LPS levels with inflammation in adipose tissue and insulin resistance that contribute to obesity and diabetes mellitus [38]. Circulating LPS levels are elevated acutely and chronically in smokers [4], and with periodontal disease [6]. In patients with severe periodontal disease, gentle mastication produces a three-fold increase in circulating LPS [39]. Subclinical levels of circulating LPS are elevated in chronic disease, including diabetes mellitus [7] and heart failure [8]. If subclinical LPS can induce cardiac fibrosis, it may contribute to the development or progression of cardiac disease in several of common conditions.

## Conclusions

Recurrent exposure to subclinical LPS is well tolerated with no signs or symptoms of disease, including normal appearance, behavior, appetite, with normal measurements of LV size and function, hemodynamics, oximetry, blood chemistries, body and heart weights. However, recurrent exposure to subclinical LPS is not benign, but has major adverse long-term consequences to induce cardiac fibrosis and decrease survival.
